# Digital ergonomics and digital work planning in university education: experiences from Germany and Austria

**DOI:** 10.1007/s41449-022-00333-7

**Published:** 2022-11-25

**Authors:** Sebastian Schlund, Christiane Kamusella, Verena Knott, Thomas Löffler, Lutz Engel, Clara Fischer, Patrick Rupprecht, Klaus Bengler, Angelika Bullinger-Hoffmann, André Kaiser, Alexander Kögel

**Affiliations:** 1 Research Unit of Human-Machine Interaction, Technische Universität Wien, Theresianumgasse 27, 1040 Wien, Austria; 2 Faculty of Mechanical Science and Engineering, Institute of Material Handling and Industrial Engineering, Chair of Labor Engineering, Technische Universität Dresden, 01062 Dresden, Germany; 3 TUM School of Engineering and Design, Chair of Ergonomics, Technical University of Munich, Boltzmannstr. 15, 85748 Garching b. München, Germany; 4 Chair of Ergonomics and Innovation Management, Technische Universität Chemnitz, 09107 Chemnitz, Germany; 5 Institute for Production and Industrial Service Management, Jade University, Friedrich-Paffrath-Str. 101, 26389 Wilhelmshaven, Germany

**Keywords:** Digital ergonomics, Digital factory, Ergonomic product and production planning, Digital human modeling, University teaching, Human factors, Digital Ergonomie, Digitale Fabrik, Ergonomische Produkt- und Produktionsplanung, Digitale Menschmodelle, Univeristätslehre

## Abstract

The publication presents an overview of the use of digital human models (DHM) in academic education at five exemplary universities in Germany and Austria. In addition to the presentation of different human models, the integration of them into the respective lectures is discussed. The teaching concepts of the individual courses of the universities, exercise examples and scenarios are presented. Experience shows that the active and independent use of digital ergonomics tools gives students pleasure and motivates them to deal intensively with complex tasks in terms of time and content. Feedback is consistently positive over all the involved lectures and universities. As a consequence of the recent Covid-19 pandemic, universities significantly increased online and blended learning. Based on the experience with the use of digital human models, the paper derives recommendations for future developments.

*Practical Relevance*

To sustain global value chains, companies are increasingly planning trans-regionally adapted products and production processes. Tools for digital ergonomics contribute to increasing competitiveness by using prospective working methods. Companies increasingly need experts with the corresponding know-how. Firmly anchoring the topic of digital ergonomics in relevant subjects of university teaching is therefore a prerequisite for this transfer of trained graduates.

## Abstract (de)

Der vorliegende Artikel gibt einen Überblick über den Einsatz von digitalen Menschmodellen (DHM) in der akademischen Ausbildung an fünf beispielhaften Hochschulen in Deutschland und Österreich. Neben der Vorstellung verschiedener Menschmodelle wird deren Einbindung in die jeweiligen Lehrveranstaltungen diskutiert. Die Lehrkonzepte der einzelnen Studiengänge der Hochschulen, Übungsbeispiele und Szenarien werden vorgestellt. Die Erfahrung zeigt, dass die aktive und selbstständige Nutzung der digitalen Ergonomie-Tools den Studierenden Freude bereitet und sie motiviert, sich mit den zeitlich und inhaltlich komplexen Aufgabenstellungen intensiv auseinanderzusetzen. Die Rückmeldungen sind über alle beteiligten Lehrveranstaltungen und Hochschulen hinweg durchweg positiv. Als Folge der jüngsten Covid-19-Pandemie haben die Universitäten das Online- und Blended-Learning-Angebot erheblich ausgeweitet. Aus den Erfahrungen mit dem Einsatz digitaler Menschmodelle werden Empfehlungen für zukünftige Entwicklungen abgeleitet.

## Background and objectives

Professional life is subject to continuous change due to increasing globalization, high competitive pressure, shortened innovation cycles and expansion of product ranges. In the last few years, the structure of the workforce and the job descriptions (proportion of women, elderly employees, staff with disabilities and impairments) have significantly changed. There is an increased focus on the added value of work performed, but also increased demands on employees and higher stress levels. In addition, Industry 4.0, which includes all stages of the product life cycle, aims at comprehensive digitization of industrial production. In this context, (partially) autonomous machines, a higher degree of individualization of the working environment and an increase in human flexibility are essential components.

All of this requires meticulous planning, well in advance of the realization phases as well as a forward-looking, proactive ergonomically optimized design of products, manufacturing processes, associated workplaces and operating equipment. Computer-based methods such as the digital factory and computer-aided product lifecycle management are increasingly gaining acceptance throughout various industries.

Digital human models coupled with ergonomics tools, which interact via simulation in a problem-solving process according to specific methods of use, are of pivotal significance. A digital human model embodies a model of the human being, which implements the respective characteristics required in the design process through software technology. Ergonomics tools encompass the analysis and evaluation methods, including their data sources, prepared for a specific ergonomics issue. (Wischniewski 2013) details the distinct advantages of digital ergonomics:

- Digital comparison of variants (without physical models); transparency in the planning process.
- Mapping of the user population and range of user characteristics via human models
- Digital evaluation of ergonomics
- Visualization support
- Reduced development time
- Early preventive safeguarding to achieve an ideal condition
- Cost savings

(Bullinger-Hoffmann and Mühlstedt 2016) cite as additional benefits improved collaboration among users from different disciplines as well as a degree of standardization of ergonomics assessments.

The relevance digital human models has significant impact on the development towards digital factory settings, whose main area of application is production planning with a digital integration of work processes. Planning aspects include e.g. sequence planning, layout creation and resource requirements planning. In addition to ergonomics and occupational safety applications, factory planning follows goals such as increasing economic efficiency through cost reduction, quality improvement and standardization. Against the background of demographic change, an overall view presupposes the early inclusion of employees with performance changes in the planning process. There are now approaches to integrate age-related performance changes into digital human models (Wirsching and Spitzhirn 2019; Spitzhirn et al. 2022b). These open up new possibilities in perspective, ability-based work design and in the generation of workplace specifications.

Accordingly, there is a growing demand for trained graduates who not only possess an overview of methods and digital tools but who can also understand and classify their mode of operation. Furthermore, they should be able to create links to fundamental ergonomics knowledge and to related disciplines and can ideally actively internalize them in an exemplary application. Considering the fact that knowledge imparted to students is implemented in practice with a delay, it is all the more crucial that corresponding teaching content is firmly integrated into the student training. This should be done with an appropriate setting, time allowance and in line with the latest state-of-the-art expertise that is geared towards the right addressees. Typically, students of engineering and economics proceed with managerial career paths in development, process and systems engineering in the areas of human factors, industrial engineering, ergonomics at the interface between technical and economic issues or in production and logistics management. The objective of university education in the field of digital ergonomics is to familiarize students with solid and future-oriented planning tools as well as their potential, to recognize the importance of virtual ergonomic safeguards and to acquire the relevant expertise. Furthermore, they acquire methodological knowledge about the sequence of steps involved in digital work planning, which aspects need to be taken into account and how appropriate tools can provide support. This will allow students to later deliberately influence ergonomic product and process development as well as workplace design in their own future work practices. Tried and tested concepts are already anchored in the curricula at different universities. These will be presented and discussed below as examples with the goal of encouraging other lecturers to devise analogue teaching content and share respective experiences.

## Integration of tools for digital ergonomics at university courses of selected institutions

### Munich approach to digital human modeling (TUM LfE 2022)

Digitalization is currently omnipresent. In order to prepare the students of *Mechanical Engineering* and *Human Factors Engineering* at the *Chair of Ergonomics, Technical University of Munich (TUM)*, for their future work and profession, it is necessary to familiarize them with the different methods and tools. The students graduate with a *Master of Science* degree after four semesters. Due to the different levels of experience and the interdisciplinary nature of the individual students, special attention must be paid to professional training in the area of teaching. Teaching at the *TUM Chair of Ergonomics* focuses on the use of different digital human models for the prospective workplace or product design. Especially RAMSIS NextGen (Human Solutions GmbH 2022) and ema Work Designer (emaWD, imk Industrial Intelligence GmbH 2022), are used for this purpose.

The ergonomics simulation software RAMSIS, as well as the follow-up version RAMSIS NextGen, can be used both as a standalone version and as a workbench in CATIA V5. As an anthropometric human model, it offers the possibility of early integration into the product development cycle. By entering parameters and boundary conditions, a probability-based posture can be calculated, and various analyses can be performed with regard to ergonomics. Reachability, as well as visibility analyses are the most frequently used modules, which are applied in the design of products and workplaces and are therefore taught in the context of teaching. RAMSIS NextGen offers more flexibility, efficiency and a better representation of the market. The user achieves results more quickly by viewing several human models (manikins) in parallel. The body shapes of the models have been improved based on real, three-dimensional scans. In addition, the possibilities of graphical visualization have been extended. The reusability of data and the improved usability of RAMSIS NextGen lead to a significantly more efficient process.

EmaWD as a standalone software enables a process module-based motion simulation for planning, designing and optimizing workflows and workplaces (Leidholdt et al. 2016, Spitzhirn et al. 2022a, b). In addition to the graphical modelling, a time-, economical- and ergonomic-oriented evaluation is possible by using the software. Different analysis methods such as standard execution time according to MTM-UAS, walking distances, proportion of value-adding activities as well as ergonomic analyzes of feasibility (reachability, visual analysis) and ergonomics and health risk assessment according to EAWS (Ergonomic Assessment Worksheet, Schaub et al. 2012) and NIOSH lifting index (Eller and Cassinelli 1994) can be used to identify economic and ergonomic problems. The EAWS screening tool enables the ergonomic assessment of static postures, action forces, load handling and repetitive activities. The result is a score and a traffic light classification into three risk areas.

Both digital human models and the associated courses complement each other. Students learn that there exists not just one human model that can be applied to any problem. On the contrary, the students are sensitized within the framework of the courses to select a suitable human model for a given simulation task. While RAMSIS NextGen is used in the RAMSIS practical course, emaWD is used in the *Ergonomics practical course *as part of a three-hour term and in the seminar *Digital Ergonomics* (cp. Table 1). In further TUM courses and application domains students can use this expertise using further DHM tools like AnyBody™ or OpenSim.

**Table 1:** Comparison of the individual courses and the use of digital human models at the TUM Chair of Ergonomics Vergleich der einzelnen Lehrveranstaltungen und des Einsatzes digitaler Menschmodelle am Lehrstuhl für Arbeitswissenschaft der TUM

Human Model	RAMSIS NextGen	Ema Work Designer	
Integration of the human model in teaching	RAMSIS practical course	Ergonomics practical course—Topic ema	Seminar Digital Ergonomics
Turnus	Winter and Summer	Winter and Summer	Winter and Summer
Teaching form	Presence/onsite	Presence/onsite	Presence or online or blended learning
ECTS	4 ECTS	4 ECTS (practical course)	6 ECTS
Hours per week	14 × 180 min	1 × 180 min	14 × 240 min
Module duration and frequency	One semester, in semester or as block	One semester, single appointment during the semester	One semester, in semester
Accessibility to systems	Computer lab TUM Chair of Ergonomics	Computer lab TUM Chair of Ergonomics	Computer lab TUM Chair of Ergonomics + Installation on student hardware
Exam	Exam with theoretical and practical part	Theory-based midterm regarding ema	Theory-based midterm, project work, report
Support material	Script, presentation, literature	Script, presentation, literature	Script, presentation, literature, videos, tutorials, problem manual

**Fig. 1:**
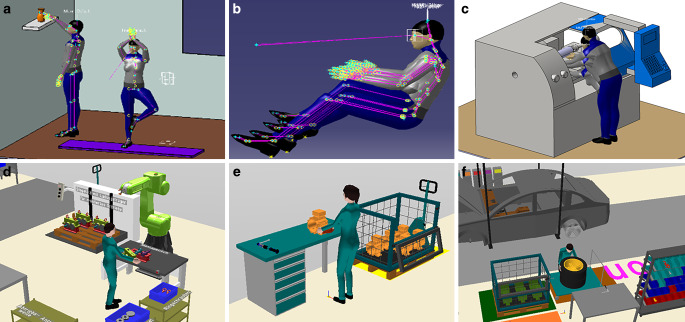
Application examples for RAMSIS in the practical course (**a**–**c**) (TUM Chair of Ergonomics, Fleischer 2022) and for emaWD in the Ergonomics practical course and Digital Ergonomics seminar (**d**–**f**) (imk automotive GmbH 2020; TUM Chair of Ergonomics, Knott 2022) Anwendungsbeispiele von RAMSIS im Rahmen und emaWD des praktischen Kurses (**a**–**c**) im Ergonomie-Praktikum und Seminar Digitale Ergonomie (**d**–**f**)

In the RAMSIS practical course, students learn to apply ergonomic product design guidelines by using RAMSIS NextGen. Using the driver’s seat of a BMW Mini as an example, students will become familiar with the possibilities of the 3D CAD human model for ergonomic interior design. They use theoretical knowledge to design a vehicle around the human being. After attending the practical course, the students will be able to understand the procedure for ergonomic vehicle interior design, remember relevant standards and apply the design of e.g. seat adjustment mechanisms of a given vehicle. Based on this, students will then be able to evaluate different products according to anthropometric aspects. Fig. 1a–c shows application examples for RAMSIS NextGen.

The *Ergonomics practical course* and the individual session on emaWD attempt to practically supplement and deepen the content of the *Ergonomics* lecture regarding the design of new workplaces. After attending the course, the students will be able to understand objective measurement and evaluation procedures in the fields of environmental and system ergonomics as well as anthropometry and to use them in a situation-related manner. As part of the topic of digital human modelling, the students learn how to use the emaWD software using the example of gear assembly. This basic knowledge helps the students to get an initial overview of the software and to familiarize themselves with the system for more detailed analyzes as part of the digital ergonomics seminar, for student research projects or later in their professional life (cp. Fig. 1d-f, below).

The seminar *Digital Ergonomics* teaches the approach of virtual planning, design and optimization of work processes, workplaces and products. After attending the course, the students are able to explain the basics of ergonomic design and evaluation and to use the emaWD software for ergonomic workplace design. Based on the example of gearbox assembly, the students will be able to transfer the contents learned to other workplace situations in the field of production/manufacturing and to carry out and assess workplace layouts according to EAWS principles. For this reason, you set up new scenarios and carry out optimizations in the ergonomic sense (e.g. assembly of an electric motor, automobile assembly (cp. Fig. 1d-f, below)).

In addition to the integration of both human models in the above-mentioned courses, the *TUM Chair of Ergonomics* supervises student research projects and theses on a wide variety of topics to deepen the application of digital human models such as RAMSIS or emaWD.

### Digital ergonomics in process and product ergonomics (TUD 2022)

The *Chair of Labor Engineering at the Technische Universität Dresden (TUD)* offers modules on *Process and Product Ergonomics* and *Work Science* for students in the *Diploma program in Mechanical Engineering*, in the *Diploma and master degrees in Industrial Engineering and Business Administration*, and in the *Diploma degree in Transportation Engineering*, where teaching content on digital a is integrated (cp. Table 2, TUD 2022).

**Table 2:** The use of digital human models at the TUD Chair of Labor Engineering Die Anwendung von digitalen Menschmodellen am Lehrstuhl für Arbeitswissenschaften der TUD

Human Model	CharAT Ergonomics	Ema Work Designer		
Degree Program	Diploma Mechanical Engineering	Diploma Mechanical Engineering	Diploma and Master Industrial Engineering and Business Administration	Diploma Transportation Engineering
Integration of the human model in teaching	Product Ergonomics and Product Safety	Industrial Engineering and Ergonomics, Part Process ergonomics	Ergonomics	Work Science
Turnus	Winter	Summer	Winter	Winter
Teaching form	Presence or online or blended learning, self-study with consultations			Presence or online
ECTS	6 ECTS (Modul)	7 ECTS (Modul)	5 ECTS (Modul)	6 ECTS (Modul)
Hours per week	8 × 180 min	10 × 180 min	10 × 180 min	2 × 180 min
Module duration and frequency	One semester, in semester	One semester, in semester	One semester, in semester	One semester, in semester
Accessibility to systems	Computer lab TUD and privately Installation on student hardware privately			Demonstration
Exam	Assignments in small groups, evaluation of the individual complex tasks and determination of a grade	Assignments in small groups, evaluation of the individual complex tasks and determination of a grade		Exam with theoretical part
Support material	Script, presentation, tutorials, videos, wikimedia system	Script, presentation, literature, tutorials, videos, Wikimedia system		Script, presentation, literature, videos, wikimedia system

The objective of the courses is to raise awareness of the significance of ergonomic task fields and to generate interest in them. Students will acquire the relevant technical and methodological expertise that will allow them to consciously and confidently shape their future professional field. They will be able to recognize the relevance and necessity of prospective working methods, in particular, via digital ergonomics validation. The teaching content includes the aspect that the two practical fields of product and process ergonomics encompass differentiated issues in their sphere of activity and that digital ergonomics tools with various functionalities have their raison d’être. Students are able to understand that digital and conventional methods, virtual and real validation complement each other in a meaningful way. They will learn that digital ergonomics tools constitute an aid in an iterative problem-solving process and that the success of their application depends on the students’ own ergonomics know-how and experience, solution strategies and their limitations. A workflow for the digital approach will be provided. The following digital tools are used:

- *Process Ergonomics*: EmaWD as a digital planning and design tool connected to the Digital Factory that supports the ergonomic planning of manufacturing processes. Variations of work systems can be designed and optimized. Manual operations as well as humanrobot interactions can be simulated, visualized and subsequently evaluated with regard to aspects of ergonomics and productivity. The digital human model embodies anthropometries according to age and gender of the different populations (e.g. German, China, Japanese, Mexican, North Americans) and has functionalities to take into account performance limitations (Spitzhirn et al. 2022b).
- *Product Ergonomics*: Digital human models like CharAT Ergonomics, prototypical in-house development of a plugin for 3ds Max, until 2012 in cooperation with VHE GmbH (Kamusella and Schmauder 2019; Kamusella et al. 2016). The human model has a kinematic skeletal structure, an anthropometric database (age, gender, nationality, proportion and somatotype) and numerous ergonomics aspects Ergotyping® tools (posture, vision, body forces, optical display devices, manual load handling). Anthropometrically distinct users of a virtual test collective can act simultaneously, whereby load effects due to causative execution conditions become traceable using ergonomics parameters and virtual imaging.

*Process Ergonomics* comprises lectures (2 weekly hours per semester) and exercises (1 weekly hour per semester). The lectures conclude with a written exam and are divided into five complexes:

- *Complex 0*: Introduction to ergonomics and fundamentals
- *Complex 1*: Anthropometric-ergonomic design
- *Complex 2*: Overview of physical loads, physiological principles, assessment procedure categories; methodology of ergonomics and risk assessment, impact on work processes
- *Complex 3*: Individual consideration of all categories of physical stress.
- *Complex 4*: Assessment of mixed loads, methodology for determining the necessity for change, relevance to processes, influences, adjusting set screws; overview of digital ergonomics tools.

These elements are supplemented by application-oriented case studies including exercises for calculation, dimensioning, analysis and evaluation according to various methodological approaches for different load scenarios. Scenes simulated using digital human models (situational workplace conditions) are also utilized, as their visualization helps to demonstrate e.g. correlations between user anthropometry and stress characteristics, both partially or in workflows.

The exercise comprises nine modules, which are structured so that teaching content is deepened through complex application-related tasks. Thus, the interconnection between paper-and-pencil methods and digital ergonomics becomes apparent:

#### *Module 0 to Module 2*:

EmaWD fundamentals: Overview of structure, background, functions, user interface and workflow; practice of navigation functions and object creation; creation of a 3D geometry model of a work system as well as the implementation of technological tasks in ema process language for a workflow (logistics and various assembly activities); analyzing the influence of errors and requirements; familiarization with functionalities for process flow control, such as intervals and fixture cycles, management of process variants; discussion of benefits and effects.

#### *Module 3 and Module 4*:

Evaluation of an ergonomically disadvantageous work setting with both paper-and-pencil methods and with emaWD; a scenario is provided for this purpose; the evaluation of individual and mixed types of physical stress is conducted with the special screening of *Leitmerkmalmethode* by the Federal Institute for Occupational Safety and Health [BAuA] (BAuA 2019) and in emaWD by EAWS ergonomics evaluation; Assessment of stress characteristics, design bottlenecks and their effects, and variants of work organization (changes in the task allocation between workers).

#### *Module 5 and 6*:

EmaWD evaluation tools: Comparison of an ACTUAL state and TARGET states in variants; comparison of EAWS evaluation, planned and specified time allowances; demonstration of possible influences for changing implementation conditions through modification of individual load characteristics using an example scenario. Demonstration of evaluation options and strategies in emaWD by using a variety of functionalities.

#### *Module 7*:

Application examples for the load type *body forces* and *forced posture*: Evaluation and discussion of various paper-and-pencil methods.

#### Module 8:

Example for the adjusting screw concept: Assessment of a stress situation, derivation of measures according to the adjusting screw concept and impact control based on specific parameters in an emaWD scene; interpretation of health hazards, of causes of stress and the identification and potential of adjusting screws.

Technical and organizational requirements for the implementation are:

- Access to scripts via OPAL (online platform for academic teaching and learning) and via Wikimedia system (https://www.ergotyping.de)
- Time-limited use of the ema software in the computer lab and privately
- Assignments in small groups
- Regular practice sessions and self-study with consultations
- Evaluation of the individual complex tasks and determination of a grade
- Provision of tutorials and instructions in videos

In the exercise *Digital Human Models* (1 SWS—semester hours per week) in the context of *Product Ergonomics and Product Safety* (2 SWS lectures), simplified and adapted application examples from industrial projects are discussed with the students. To this purpose, a structured course of action is taught and worked through step by step in ergonomic subtasks. The CharAT Ergonomics software is available to students in the computer lab and privately for a limited period of time. Project tasks are worked on in small groups in on-site exercises and self-study with consultation and then the grade is determined.

Assignments include: The creation of an animation scene to elucidate human model configuration and movement in the context of product handling (introductory example); dimensional design of a multifunctional armrest for mobile machines taking into account user anthropometries; anthropometric design of a tiler’s table; ergonomic requirements for choosing operating elements of an armrest control console; evaluation and design of visibility conditions at a test workstation.

**Fig. 2:**
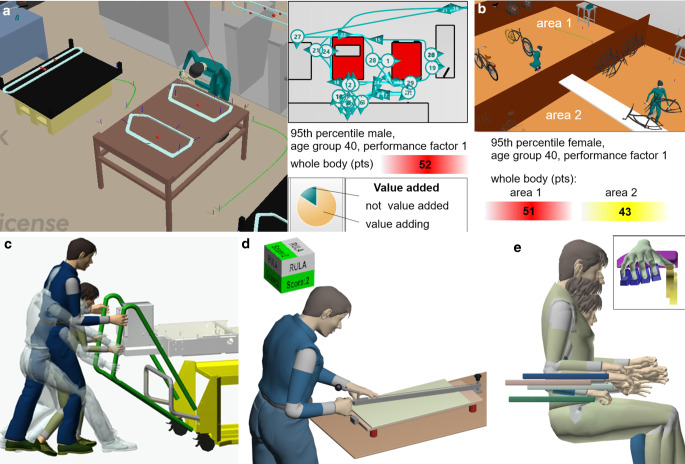
Application examples for emaWD version 1.9.7 in the exercise Process Ergonomics (**a**, **b**) and for the exercise Digital Human Models (Product Ergonomics) with CharAT Ergonomics (**c**–**e**) Anwendungsbeispiele von emaWD Version 1.9.7 in der Übung Process Ergonomics (**a**, **b**) und für die Übung Digital Human Models (Product Ergonomics) mit CharAT Ergonomics (**c**–**e**)

Fig. 2 shows application examples for emaWD in the exercise *Process Ergonomics* (Fig. 2a, b) and for the exercise *Digital Human Models* with CharAT Ergonomics (Fig. 2c–e).

### Digital simulation of ergonomics and robotics (TU Wien 2022)

At the *Institute of Management Science*, the research group *Human-Machine Interaction* develops various industrial demonstrators for human-machine interaction, mostly at *TU Wien Pilot Factory*. In the context of designing human-oriented workplaces, the focus of the research group is on the use of assistance systems such as collaborative robots. The digital planning of robotic cells is a classic application of factory planning, but when using cobots, the consideration of human parameters and behaviour is indispensable (Fischer and Schlund 2021).

Against this background and with particular emphasis on ergonomics in mutual human-robot work systems, a lecture entitled *Digital Simulation of Ergonomics and Robotics (DSER)* was initiated in 2020. The lecture aims at master students of Mechanical Engineering and Industrial Engineering. The software tools emaWD (imk Industrial Intelligence GmbH 2022) and Tecnomatix Process Simulate (Siemens AG 2022) with the respective human models are used to simulate workplaces that have been designed within various research projects. Well established ergonomics risk assessment methods (NIOSH and EAWS) can be used and attribute scores for work postures, forces, loads and in EAWS also additional extra points for specific tasks, such as unergonomic wrist positions. The scores are summed up, in order to evaluate the task by comparing it to a final score (Schaub et al. 2012).

**Fig. 3:**
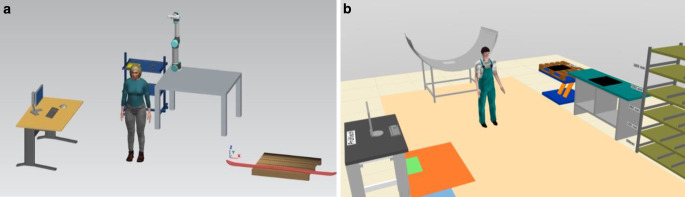
Initial scenario of use case **a** ski binding assembly in Process Simulate **b** Fan cowl in emaWD version 2.0 Ausgangsszenario des Anwendungsfalls **a** Skibindungsmontage in Process Simulate **b** Fan cowl in emaWD version 2.0

Students learn about the role of ergonomics in manufacturing, use various analysis tools for its evaluation and employ assistance systems for ergonomic improvements. Furthermore, they get to know digital human models and process simulation tools theoretically and practically through the implementation and improvement of industry-like use cases. The DSER course of 3 ECTS covers a period of six weeks and was delivered in an online blended learning mode due to the Covid-19 pandemic, consisting of distance self-learning and online live lectures. The communication software Microsoft Teams is used for the online lectures. After an online organizational meeting, the students learn the theoretical content in a self-study phase. A course on TUWEL, the TU Wien online learning platform, provides access to e‑lectures, slides and non-graded self-assessment tests. This theoretical part covers a period of three weeks, in which the students can work time flexibly on the documents. In order to proceed to the practical part, participants perform a graded multiple-choice test, which has to be passed in order to proceed with the practical part of the lecture. This part starts with a virtual live lecture with focus on the simulation software. Hands-on practical examples deepen the knowledge. After an introduction to the user interface, the first steps of the use case simulations are carried out. Furthermore, there is usually at least one topically related guest lecture from industry. Afterwards, students have three weeks to work in groups of two on the simulation and improvement of a chosen use case: *human-cobot interaction* or *fan cowl assembly*. Program files with the initial scenarios of the use cases are provided in order to ensure that everyone works with the same arrangement (cp. Fig. 3). The cobot use case illustrates a part of the assembly of a ski binding. Followed by the instructions on a PC-terminal, the human model picks up the ski from a pallet, puts it on the working table and screws together the binding components after the robot has placed them correctly on the ski. The second use case represents a manual fan cowl assembly. A fan cowl is an aircraft turbine housing that is manufactured layer by layer by mounting carbon mats. In the initial situation, the mats are placed on the pallet in an ergonomically unsuitable position and should be placed on the assembly object, according to the instructions of the PC-terminal.

Within a group, both simulation tools are distributed between the two team members. For each scenario, a help document, that introduces the user interface and gives step-by-step instructions for setting up the simulation, is provided via TUWEL by the supervisors. Students also can use videos to see what the final representation of the use case should look like. Furthermore, the students can use the ema help catalogue and the documentation of Siemens Learning Advantage, respectively. After a joint set-up of the initial scenario, the students develop and evaluate ergonomic improvements and implement them separately. In order to identify improvements, multiple analyses, such as path, time and ergonomics assessment are recommended. Within the project, mandatory consultation meetings to discuss intermediate results are carried out. It turned out that the use of an online collaboration tool facilitates the discussion, as the software allows to share control of the screen to solve problems directly in the programs of the students. In the closing lecture, the students present their group results as well as their experiences with the two simulation programs with special emphasis on the use of DHM.

**Fig. 4:**
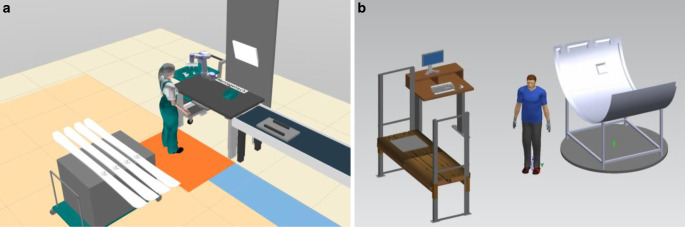
Best practice examples **a** ski on conveyor belt in emaWD version 2.0 **b** fan cowl on turntable in Process Simulate Beispielhaftes verbessertes Szenario **a** Ski auf Förderband in ema WD Version 2.0 **b** Fan cowl auf Drehtisch in Process Simulate

Two student examples of ergonomics improvements are shown in Fig. 4, one for each use case. For both scenarios, the student groups raised the starting positions for the material, for example by replacing the pallet in order to avoid unergonomic lifting. In the scenario of the ski use case, a conveyor belt was installed that moves the ski directly to the assembly position, cp. Fig. 4a). Furthermore, a display for work instructions was mounted on top of the workplace. Besides significantly improving the body postures, from unfavourable 53.5 EAWS points (‘red’ region of high ergonomics risk) to suitable 13.5 points, the walking distance was reduced to less than half. Similar results were achieved in the shown fan cowl scenario, cp. Fig. 4b). It is realized by a U-shaped assembly station, where the instruction terminal and material pallets are placed on tables of adjustable height. Furthermore, the fan cowl is mounted on a turntable that rotates towards favourable working positions, so that the employee can always walk straight ahead after picking up the carbon mat and mounting it on the component.

The student’s grades consist of the results of the online test (10%), presentation (30%) and exercises (60%). Assessment criteria of the exercises include structure and functionality of the use case and ergonomics improvements. Presentation, ergonomic analysis and comparison of the simulations are evaluated as well. Students have the opportunity to further develop their skills in the use of digital human models as part of a seminar or master thesis, after the course. For future courses, the ergonomic parts of the lectures will be expanded by a practical EAWS lecture, where students learn to use EAWS hand sheets as well as automatic evaluation with the help of motion tracking. In addition, the focus on robotics simulation will also be expanded. However, the online self-study phase will remain online. The same applies to status meetings, also because the screen control in MS Teams has proven as a very helpful tool for quick and effective problem solving.

### Digital simulation in industrial engineering (Jade Hochschule 2022)

Digital examination of products within the framework of an underlying virtual production prior to real implementation embodies an immense trend for the future. Changes arising from this trend are not solely influencing industry practice, but also the state-of-the-art design of teaching. Simply working through course contents utilizing slide decks without experimental components does not lead to a promising transfer of knowledge anymore.

But how can knowledge from industrial engineering be conveyed vividly and practically while due to Covid-19 restrictions there is no possibility to experiment in real factories? Derived from this question, a newly structured teaching concept is presented, which finds its initial application during the module *Industrial Engineering* as part of the master’s program *Wirtschaftsingenieurwesen* at *Jade University of Applied Sciences*.

This course aims to enable students to independently analyze, design and improve micro and macro work systems from efficiency and humanization perspectives as realistically as possible utilizing digital tools like emaWD (imk Industrial Intelligence 2022) and visTABLE (plavis 2022).

To enable the students to enter the subject of Industry 4.0, additionally, the software OPC Router (inray 2022) is deployed. The overall student’s workload accumulates to 240 h, of which 72 h are allocated for contact study and 168 h for self-study. The lecture is offered annually with 4 SWS and is considered to be mandatory with 8 ECTS.

**Fig. 5:**
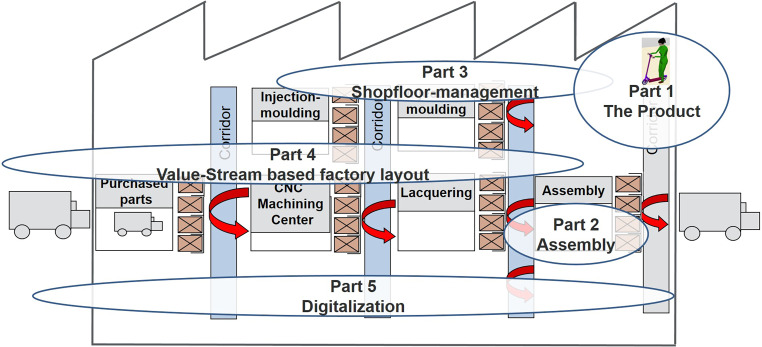
Work Packages for Virtual Design of E‑Scooter Production Arbeitspakete für den virtuellen Entwurf einer E-Scooter Production

The idea of this one-semester teaching concept is that the instructor acts as a fictional customer, who requests students to submit an offer in the form of a concept for the production of a product like an e‑scooter. Within this concept, students work in teams of two and are required to compete with other student teams across the module to prove themselves as potential product suppliers. Instead of the lowest price product prioritization, the primary focus is the fictional customer’s desire for a transparent concept presentation for value-added service production developed by the teams over the course of the semester. Progress is continuously presented by the teams and based on the value stream for product manufacturing which is illustrated in Fig. 5.

Concept development starts for the students in Part 1 by getting to know the product and the customer requirements. Subsequently, product, process and resource data suitable for assembly are developed in cooperation with the fictional customer. In Part 2, the concept for product assembly in the form of a manual flow line with integrated human-robot interaction is digitally modelled utilizing the emaWD software. The emerging assembly embodies the pacemaker within the overall process of value creation and is therefore considered as the entry point for process representation. The process times determined deploying MTM methods incorporated in the emaWD should be shorter than the demand-dependent takt time and the ergonomic values determined deploying EAWS should be in the range with a low risk of biomechanical overload. Levelling the overall flow is an additional challenge.

**Fig. 6:**
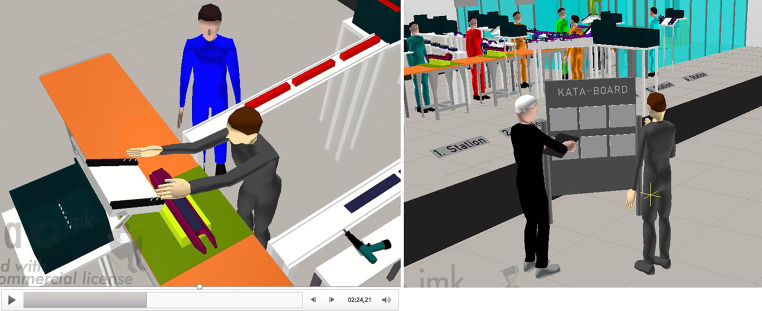
TWI Role Play Video and Coaching KATA Mentor/Mentee Utilizing emaWD version 1.9.7 TWI Rollenspiel Video und Coaching KATA Mentor/Mentee unter Verwendung von emaWD Version 1.9.7

In Part 3, students practice in teams how leadership is performed on the shopfloor level with e.g. Toyota KATA (Rother 2010) utilizing previously digitally developed assembly. Thereby a continuous improvement process is created in the direction of further value creation. Furthermore, a concept for the training of employees at the assembly line applying TWI (Training Within Industry) is developed, which is practiced at the virtual assembly line (cp. Fig. 6). The students’ ability to apply TWI must be demonstrated in a short video that shows the simulation of the assembly from emaWD and a role play between mentor and mentee.

With the utilization of preparatory work from Part 1 (process and resource data), the entire value stream is finally designed in Part 4 for all components of the product, which are manufactured in-house. The virtual representation of the value stream, including layout planning and material flow analysis, is performed via the visTABLE software. By expanding the ‘classic’ value stream mapping by additional elaboration of information logistics (Meudt et al. 2017), the students learn to identify key performance indicators within the production process, which are to be digitized in Part 5. During this last part of the overall concept, the students perform Industry 4.0 experiments and practice digitization of processes using smart sensors, cloud technologies and the OPC Router communication software.

For the application of the presented overall teaching concept, for each semester an individual product or demonstrator is selected and CAD data of the product to be assembled is prepared in detail. During the semester, students are taught theoretical foundations by the instructor, receive content through tutorials held by trained lab engineers and get access to explanatory videos on the integrated software. Students can immediately put newly acquired knowledge into action, because virtually created assembly concepts as well as the entire production can be modified experimentally. Through the role plays, the students practice managing employees and change processes, while learning leadership skills that would not be achieved solely through lectures. Utilizing tools such as Virtual Reality (VR) and Augmented Reality (AR), students are enabled to virtually ‘walk through’ the assembly and the factory. Via virtual collaboration, students can work simultaneously on the same factory in a virtual space. To evaluate the concept, the students must create a written elaboration and explanatory videos. In the videos, the students present their demonstrator and the respective concept of industrial production. The team who presents the overall best concept according to customer requirements receives the (fictional) order and a corresponding very good grade.

During the course, the master’s students deal with individual topics Part 1 to Part 5 whereby many activities can be performed independently and digitally. An additional learning effect is achieved through regular presentations and discussions of partial results of the topics in front of and within a group. The module is also characterized by the fact that the event is held in the form of a competition, which encourages students’ ambition. In retrospect, a master’s student evaluates the module as follows: “My experience is that the module is very practical and includes many digital tools that are valuable and can be used directly for planning tasks in companies.” A reduction in students’ workload is possible by a future concept which focuses on an already existing production (brownfield planning). To further improve the interfaces between the software packages, emaPD (imk Industrial Intelligence GmbH 2022) will also be used additionally in the future for layout planning and material flow analysis.

### Digital ergonomics in advanced manufacturing (TU Chemnitz 2018)

The Chair of Ergonomics and Innovation Management at the Technische Universität Chemnitz is involved in research and teaching on digital human models for quite some time (Bullinger-Hoffmann and Mühlstedt 2016; Kaiser et al. 2020; Kaiser and Bullinger 2020). In terms of teaching, the chair customarily offers single tutorials on digital human models within courses such as *Arbeitswissenschaft* and *Arbeitsanalyse und Arbeitsgestaltung*. These courses are part of various degree programs. Within the tutorials, students gain a general overview of purposes, concepts and types of digital human models. They also find opportunities to try out digital human model software. For example, they can manipulate given manikins and scenes, assess and—to a suitable extent—improve existing models of workplaces.

Since 2018, the chair has participated in the Master’s program *Advanced Manufacturing* by means of the elective course *Digital Ergonomics*, where digital human models make up a large part of the course. The course is held in English. Teaching forms are seminars (90 min per week) and exercises (90 min per week). The student’s workload amounts to 150 working hours. Five credit points are awarded.

Qualification objectives of *Digital Ergonomics* are that students can apply the principles and rules of ergonomics analysis and work design with the help of selected digital tools. They should be able to reflect on the potential and limits of digital ergonomics.

In the course, various self-learning material for ergonomic analysis and ergonomic work design is provided. On this basis, methodological skills are deepened in seminars and exercises in order to apply ergonomic concepts with the help of advanced digital tools. The seminars focus on:

- Basic concepts of ergonomics work analysis (e.g. stress and strain)
- Selected standards and methods of ergonomics work analysis and work design as for
  - Anthropometry (DIN EN ISO 7250 (2017); DIN 33402 (2020)),
  - Weight handling, physical strength, posture (Ergonomic Assessment Worksheet),
  - Target times (Methods-Time Measurement)
- Modelling with digital human models (history, concepts, types, implementation, analysis functions, future trends)
- Virtual reality and ergonomics
- Motion capturing

**Fig. 7:**
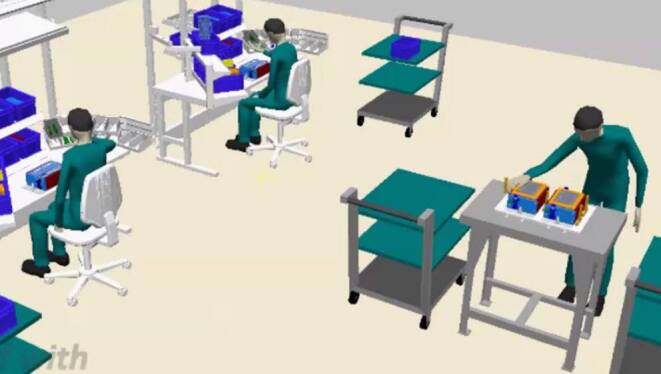
Work system model of a battery assembly line using emaWD version 1.9.4 Simulationsmodell einer Batterie Montage Linie unter Verwendung von emaWD Version 1.9.4

Subject to the exercises and the homework is a case study—the analyses and redesign of a work system—consisting of:

- 3D modelling of a work systems with Trimble SketchUp Pro (cp. Fig. 7, a factory hall, two sitting and one standing workplaces have to be modeled)
- Process modeling of manual handling tasks with a cycle time of 130 s with emaWD
- Anthropometric design of a workplace (e.g. calculation of the table height)
- Ergonomic and time analyses with emaWD
- Deriving ergonomics improvement measures

**Fig. 8:**
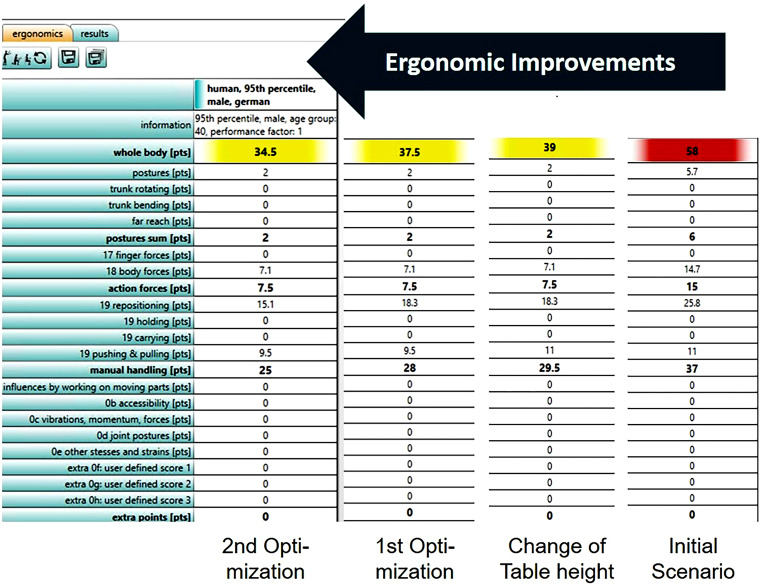
Example of a student’s presentation for results of ergonomic improvements with emaWD Beispiel aus einer Studentenpräsentation, mit den Ergebnissen der Ergonomieverbesserung mit emaWD

Students have to supply five recorded practical achievements as part of homework, and a report of the case study including developed optimizations. The final event is an oral presentation and discussion of the results of the case study (cp. Fig. 8).

Usually, exercises were held at the university’s PC pool using educational licenses for ema Work Designer and SketchUp Pro. Due to the Covid-19 pandemic, students had to attend remotely using their private computers or logging into virtual computers provided by the university. Appropriate software licenses were provided as well.

In the course described the use of digital human models in teaching shows the following advantages:

- The immersive and interactive content offers opportunities for a better understanding of work systems even for students without industrial experience.
- Students receive direct feedback (ergonomic scores and cycle time) on their modifications of the workplace design even when no supervisor is present.

On the other hand, there are disadvantages and challenges:

- The expenditure for preparation of the course materials, for the individual support and for the exams is increased compared to other courses.
- An advanced technical infrastructure is required.
- The results of the ergonomics assessments and time analysis are difficult to compare due to possible variations in the underlying 3D environments modelled by the students themselves.
- Some students tend not to reflect on the results of software-based assessments.

However, the increasing number of participants shows the success of the (elective) course.

## Recommendations for the use of digital human modeling in university education

### Lessons learned

Digital simulations with the use of digital human models have the potential to improve teaching in various engineering disciplines such as mechanical engineering, industrial engineering and management and product development as demonstrated in training processes in the industry (Dennerlein et al. 2015; Spitzhirn et al. 2017). Digital simulations enable various scenarios, different planning alternatives and the setup of complex work systems.

The following advantages become evident throughout the use of digital ergonomics tools, especially in university teaching:

- The immersive and interactive content offers opportunities for a better understanding of work systems even for students without industrial experience.
- Students receive direct feedback (ergonomics scores and cycle time) on their modifications of the workplace design even when no supervisor is present.
- Digital tools enable integrated and continuous workflows from the digital creation of the work system, through simulation of the work activities, to ergonomics assessment and design of the work process.
- Competence-oriented learning becomes possible through the application of theoretical knowledge in the problem-solving process.
- Depending on the didactic concept, teamwork can be promoted by group tasks.
- In some cases, no or little CAD knowledge is required, as 3D environments can be created with the integrated CAD library or with substitute geometries.
- Dependencies and interactions become understandable (e.g. anthropometrically determined virtual user groups; direct change effects on health risk, added value, process time).
- Advantages of virtual design and analysis methods such as rapid modification and feedback can be shown.

Using digital human models for academic teaching purposes still bears some inconveniences such as the time-consuming setup and upgrade routines before each course and, in some cases—especially regarding the integrated software suites—a complex learning procedure for the program itself. At the moment the preparation and supervision effort for the teaching staff is still high, as individual support is required for problem-solving in dealing with the software, depending on the previous knowledge of the students. The results of the ergonomics assessments and time analysis are difficult to compare due to possible variations in the underlying 3D environments modelled by the students themselves. However, the experiences in the courses also help to feedback on important aspects, findings, advantages, and disadvantages to the manufacturers of digital human models. The manufacturers can benefit from these statements, weigh them up, and reflect them in revisions. Universities and research institutions not only focus on the result of the analysis but also on the usability of software and intuitive application, in order to keep the load on the user of the software as low as possible.

Experience shows that the active and independent use of digital ergonomics tools gives students pleasure and motivates them to deal intensively with complex tasks in terms of time and content. Feedback is consistently positive over all the involved lectures and universities. Students develop an interest in deepening ergonomics subject areas in the further course of their studies (e.g. in the form of seminar papers and theses) and internalizing them in their future professional lives. The sensible dovetailing of paper-and-pencil methods or theoretical principles and digital ergonomics tools are perceived more strongly.

Furthermore, the automated evaluation and optimization assessment focus more on the results and less on the methodological foundation of the evaluation. Thus, additional theoretical input—e.g. basic principles of productivity and ergonomics evaluation—is still necessary. For example, prior learning of paper-and-pencil methods is recommended. Last but not least, digital simulations only provide modelled instances of reality. Despite various simulations and tests, some impacts of work system design can be perceived in reality only. Especially for the use in SMEs and the involvement of shop floor staff, tangible, hands-on prototypes often provide a feasible alternative for early participation and commitment of people that are not familiar with digital planning tools.

### Blended learning

As worldwide pandemics forced the use of digital learning, the use of digital human models has experienced a boost in relevance for teaching, but also for implementation purposes in industry. Digital tools, such as human models have proven to be valuable alternatives to classroom teaching and physical prototypes. From a short-term perspective, they even replaced traditional approaches. Based on the experience, but also the inconveniences of interaction and commitment in online teaching settings, we expect a stronger trend towards hybridization of real and digital tools in education over the long term. Lessons learned over all the presented approaches show a preference towards project-based, multi-modal learning settings with well-chosen tool use. These experiences align well with the results of industrial up- and reskilling approaches (Hader et al. 2022). The opportunities to use synchronous and asynchronous learning settings have been well-received by the students as well as a return to work-based learning infrastructures such as learning factories (Komenda et al. 2021). Consequently, digital simulations are considered as an integral part of future skill sets in work system planning.

### Digital twins and cognitive systems

Beyond education and learning purposes, we expect a growing emphasis on digital human models within the ongoing discussion on further integration of physical virtual characteristics of products, processes and services. Various sensors, sensor fusion, and wireless data transmission enable the creation of digital twins as digital representations of objects and product-services (Stark and Damerau 2019). In order to promote the design of work systems according to the principles of socio-technical systems the further integration of human parameters, behaviour and requirements will be of crucial importance. Therefore, existing approaches need to be improved in terms of standardized data interfaces, transparent algorithms, further analysis functions, a realistic representation of the human and real real-time synchronization with the physical work system. For digital twins used for (longer-term) planning, generic human models with typical percentile characteristics and standard performance may be sufficient. Especially if the digital twins are to be synchronized with physical production in (almost) real-time for the purposes of operational planning and control, digital human models must be more customizable (e.g. individual qualifications, skill limitations, availability). In this regard, the first steps have been taken with regard to age-related ability changes (Wirsching and Spitzhirn 2019; Spitzhirn et al. 2022b). Recent developments in sensor technology allow performant use of human activity recognition and adaptable work systems (Schlund and Kostolani 2022). Consequently, the traditional planning process of work system design, planning and use might be developed into a dynamic on-site improvement of an adaptive work system, whereas possible adjustments are simulated in parallel via a digital twin including a digital human model.
